# On the Excretion of Phosphoric Acid by the Kidneys

**Published:** 1857-09

**Authors:** William A. Hammond

**Affiliations:** Assistant Surgeon U. S. Army; U.S.A.


### ART. III.—*On the Excretion of Phosphoric Acid by the Kidneys.* By WILLIAM A. HAMMOND, M.D., Assistant Surgeon U. S. Army.

THE amount of phosphoric acid excreted through the urine, has recently been specially investigated, under various physiological conditions, by Breed,* Winter,† Mosler,‡ Bencke,§ and Böcker,|| and has also been incidentally studied by many other physiologists. Experiments relative to tissue-metamorphosis are, however, always of interest, and I shall therefore offer no apology for going over, in part, the ground previously traversed by other investigators.

The experiments detailed in this paper were all performed upon myself,

* Annalen der Chemie. Band 78, Heft 2.

† Beiträge zur Kenntniss der Urinabsonderung bei Gesunden, Inaug. Abhandlung. Giessen, 1852.

‡ Beiträge zur Kenntniss der Urinabsonderung, Inaug. Abhandlung. Giessen, 1853.

§ Archiv für wissenschaftliche Heilkunde, B. 1, H. 4.

|| Archiv für wissenschaftliche Heilkunde, B. 2, H. 2.

and had reference principally to the determination of the normal amount of phosphoric acid excreted under ordinary physiological conditions, and to the variations induced by strong bodily exercise and the internal administration of phosphate of soda. The quantity and specific gravity of the urine, and the weight of the body, were also ascertained.

The day was divided into three periods. The first, or morning, commenced at 7 A.M., and ended at 1 P.M.; the second, or evening, extended from 1 P.M. to 10 P.M.; and the third, or night, from 10 P.M. to 7 A.M. The urine of each of these divisions was accurately measured, its specific gravity and amount of phosphoric acid ascertained, and the weight of the body, at the end of each period, carefully determined.

The methods employed were as follows:

The *quantity* of urine was measured in a cylindrical glass vessel, holding 100 cubic centimetres, and graduated to half a cubic centimetre. For convenience, the quantity of urine is expressed in cubic centimetres instead of fluid ounces.*

The *specific gravity* was determined by means of the specific gravity bottle, and weighing in a delicate balance. The so-called urinometers, sold in the United States, are, according to my experience, absolutely worthless for obtaining results of even moderate accuracy.

The *amount of phosphoric acid* was ascertained by the volumetric method with perchloride of iron. As this process has not yet been fully laid before the profession in this country, it may be expedient to describe it in detail as originally proposed by Liebig and employed by Breed.†

"This process consists simply in the addition to the urine of a titrited solution of perchloride of iron, till a filtered sample of the mixture yields a blue color with ferrocyanide of potassium. It depends upon the fact that a fluid (whether neutral or acidified with acetic acid), which contains phosphoric acid, gives an insoluble precipitate with a fluid containing peroxide of iron.

"The solution of perchloride of iron is best prepared by dissolving 15·556 grammes of iron in nitro-hydrochloric acid, carefully evaporating to dryness in a water-bath, so as to expel the excess of acid, and dissolving the solid residue in 2000 cubic centimetres of water. One cubic centimetre of this solution precipitates ten milligrammes of phosphoric acid.

"Instead of such a solution of perchloride of iron, one of indefinite concentration may be employed, the strength of which has been previously ascertained by titrition with a solution of phosphate of soda containing a known quantity of phosphoric acid. In each case the solution of perchloride of iron must be free from chloride of iron.

"Should the urine, whose proportion of phosphoric acid is to be deter-

* Thirty-three cubic centimetres are about equal to one fluid ounce. † Op. cit.

mined, be of alkaline reaction through the decomposition of its urea, it is probable that a part of the phosphoric acid has already been precipitated in union with lime or magnesia. In this event it is necessary to redissolve the precipitate by the addition of a few drops of nitric acid.

"The urine is measured and well agitated. A definite quantity (100 cubic centimetres or more), is, by means of a pipette, placed in a beaker-glass, and a solution of acetate of soda (a considerable amount when nitric acid has been added) and acetic acid mixed with it. Then, by means of a burette, the perchloride of iron is added, and the urine frequently tested, to determine whether or not the whole of the phosphoric acid has been precipitated, till a trace of an excess of iron has been added. In order to ascertain when this last is the case, a piece of filtering paper, moistened with a solution of ferrocyanide of potassium, is spread out upon a white porcelain dish (or on a glass plate over white paper), and a folded piece of filtering paper pressed against it by means of a glass rod on which a drop of the urine hangs. If an excess of solution of iron has been added, a blue color appears in three or four seconds; the quantity of perchloride of iron necessarily employed is noted. In a similar manner two other portions of the urine should be proceeded with. If the results agree, it is calculated how much of the solution of iron would have been necessary for the whole quantity of urine, and the amount of phosphoric acid indicated by this quantity of the reagent is that which the total amount of urine contains."

The acetate of soda and acetic acid may be conveniently employed by making a solution of the salt in the proportion of twenty grammes to 160 cubic centimetres of distilled water, and adding forty cubic centimetres of the concentrated acid. Ten cubic centimetres of this solution contain one gramme of acetate of soda, and two grammes of acetic acid. In ordinary cases, twenty cubic centimetres are used for every 100 cubic centimetres of urine. Where the urine is alkaline, and it has been necessary to dissolve any resulting precipitate of phosphates with nitric acid, as before specified, the quantity of the above solution to be employed should range from twenty to forty cubic centimetres, according to the amount of nitric acid necessarily added.

By the foregoing described method, results are obtained of at least as great accuracy as by precipitation and subsequent weighing, and with much less expenditure of time and labor.*

* The decimal system of the French is so much more convenient than our own, in chemical manipulations, and is now so generally employed in such operations, that I have not deemed it necessary to reduce the weights and measures of the foregoing process to the English standard. A little reflection, and an acquaintance with the general principles of chemistry, will enable those who wish it, to perform all the described manipulations with the domestic weights and measures.

The weight of the body was ascertained by means of a balance capable of turning with ·01 pound when loaded with 250 pounds.

During the experiments I breakfasted at 7 A.M., lunched at 1 P.M., and dined at 5 P.M. At breakfast I ate five ounces of beefsteak, eight of bread, one half ounce of butter, and ten grains of salt, and drank six ounces of strong coffee, containing two drachms of cream and two of white sugar. At luncheon I ate three ounces of cold roast beef, six of bread, two drachms of butter, and twenty grains of salt. At dinner I took six ounces of strong beef soup, eight of roast beef, four of potatoes, four of bread, two drachms of butter, and half a drachm of salt, and drank four ounces of coffee. In addition to this food I drank, at each meal, twelve ounces of water, and six immediately before going to bed.

In the twenty-four hours, therefore, I ingested sixteen ounces of beef, eighteen of bread, six of soup, four of potatoes, one of butter, one drachm of salt, two of cream, and two of sugar, and drank ten ounces of coffee and forty-two of water.

This diet does not differ greatly from that followed during former investigations of the effects of alcohol and tobacco upon the human system.*

The standard amount of physical exercise (divided as equally as possible between the morning and evening periods of the day), consisted in walking about one thousand yards.

The mental exercise consisted of reading and study from 9 A.M. to 1 P.M., chemical investigations from 2 P.M. to 5 P.M., and reading (generally of a light character), from 8 P.M. to 10 P.M., at which latter hour I retired to bed. The remaining active hours of the day were passed in necessary duties, recreation, &c. I awoke at about 7 A.M., and consequently slept about nine hours. The fæces were evacuated at a few minutes after 7 A.M., and the urine at the termination of each period.

The quantity of urine is given in cubic centimetres, the phosphoric acid in troy grains, and the weight of the body in avoirdupois pounds.

### I.

### STANDARD SERIES.

This series continued eight days, under the conditions already specified. The results are contained in the accompanying table:

* American Journal of the Medical Sciences, October, 1856.

TABLE I.

| Date. | Morning (6 hours). | | | | Evening (9 hours). | | | | Night (9 hours). | | | | Whole Day. | | | |
|---|---|---|---|---|---|---|---|---|---|---|---|---|---|---|---|---|
| | Quantity of Urine. | Specific Gravity. | Phosph. Acid. | Weight of Body. | Quantity of Urine. | Specific Gravity. | Phosph. Acid. | Weight of Body. | Quantity of Urine. | Specific Gravity. | Phosph. Acid. | Weight of Body. | Quantity of Urine. | Specific Gravity (Mean). | Phosph. Acid. | Weight of Body (Mean). |
| April 2d, | 427·5 | 1022·51 | 20·978 | 196·50 | 667·8 | 1025·30 | 25·103 | 196·72 | 498·3 | 1024·81 | 14·090 | 196·55 | 1593·6 | 1024·20 | 60·171 | 196·55 |
| " 3d, | 441·6 | 1023·04 | 21·820 | 196·42 | 825·5 | 1022·17 | 28·251 | 196·59 | 345·7 | 1025·43 | 10·241 | 196·50 | 1612·7 | 1023·54 | 60·312 | 196·50 |
| " 4th, | 378· | 1024·35 | 17·531 | 196·41 | 594·8 | 1025·21 | 31·518 | 196·53 | 685·4 | 1023·62 | 16·356 | 196·44 | 1658·2 | 1024·39 | 65·405 | 196·46 |
| " 5th, | 426·7 | 1023·46 | 22·430 | 196·48 | 500·6 | 1024·37 | 25·211 | 196·75 | 524·8 | 1024·12 | 10·287 | 196 61 | 1452·1 | 1023 98 | 57·928 | 196·61 |
| " 6th, | 504·2 | 1021·59 | 15·260 | 196·30 | 515·4 | 1025·11 | 19·514 | 196·45 | 438·9 | 1024·67 | 15·785 | 196·40 | 1458·5 | 1023·79 | 50·559 | 196·38 |
| " 7th, | 450·1 | 1022·41 | 19·389 | 196·15 | 492·7 | 1025·04 | 22·301 | 196·27 | 401·5 | 1025·02 | 16·424 | 196·24 | 1344·3 | 1024·15 | 58·114 | 196·22 |
| " 8th, | 518·5 | 1021·07 | 24·758 | 195·89 | 486·9 | 1025·49 | 20·427 | 196·01 | 454·2 | 1024·35 | 17·438 | 195·94 | 1459·6 | 1023·63 | 62·623 | 195·94 |
| " 9th, | 412·4 | 1021·58 | 22·507 | 195·95 | 514·6 | 1024·21 | 25·407 | 196·10 | 489·6 | 1023·59 | 10·527 | 196·04 | 1416·6 | 1023·12 | 58·441 | 196·03 |
| Average, | 444·8 | 1022·50 | 20·584 | 196·26 | 574·8 | 1024·61 | 24·729 | 196·44 | 479·8 | 1024·45 | 13·893 | 196·34 | 1499·4 | 1023·97 | 59·194 | 196·33 |

An examination of the foregoing table shows the following facts:

1st. The quantity of urine excreted was highest for the evening period, next highest during the night, and lowest in the morning.

2d. The evening urine was of highest specific gravity, the night urine somewhat less, and the morning urine the lowest.

3d. The evening urine contained the greatest amount of phosphoric acid, the morning urine the next largest, and the night urine the smallest.

4th. The weight of the body was greatest at evening, next greatest at night, and smallest in the morning.

The following table, showing the amount of urine and of phosphoric acid passed per hour during each period, the proportion of phosphoric acid in 1000 c. cen. of urine, and the quantity of urine and phosphoric acid excreted for each pound weight of the body, will tend further to elucidate the subject.

TABLE II.

| Date. | Morning (6 hours). | | | | | Evening (9 hours). | | | | | Night (9 hours). | | | | | Whole Day. | | | | |
|---|---|---|---|---|---|---|---|---|---|---|---|---|---|---|---|---|---|---|---|---|
| | Urine per hour. | Phosph. Acid per hour. | Phosph. Acid in 1000 C.CM. of Urine. | Urine per pound weight of the Body. | Phosph. Acid per pound weight of the Body. | Urine per hour. | Phosph. Acid per hour. | Phosph. Acid in 1000 C.CM. of Urine. | Urine per pound weight of the Body. | Phosph. Acid per pound weight of the Body. | Urine per hour. | Phosph. Acid per hour. | Phosph. Acid in 1000 C.CM. of Urine. | Urine per pound weight of the Body. | Phosph. Acid per pound weight of the Body. | Urine per hour. | Phosph. Acid per hour. | Phosph. Acid in 1000 C.CM. of Urine. | Urine per pound weight of the Body. | Phosph. Acid per pound weight of the Body. |
| April 2d, | 71·2 | 3·466 | 48·67 | 2·12 | ·157 | 74·2 | 2·799 | 37 72 | 3·39 | ·122 | 55·3 | 1·565 | 28·30 | 2·53 | ·071 | 66·4 | 2·507 | 37·75 | 8·11 | ·306 |
| " 3d, | 73 6 | 3·636 | 49·40 | 2·24 | ·105 | 91·7 | 3·139 | 32·05 | 4·10 | ·143 | 36·2 | 1·138 | 31·16 | 1·75 | ·052 | 67·4 | 2·513 | 37·28 | 8·21 | ·307 |
| " 4th, | 63·0 | 2·921 | 46 36 | 1·95 | ·089 | 66·1 | 3·502 | 52·98 | 3·02 | ·163 | 76·2 | 1·150 | 15·09 | 3·48 | ·083 | 69·1 | 2·725 | 39·47 | 8·44 | ·338 |
| " 5th, | 71·1 | 3·405 | 47·88 | 2·12 | ·114 | 55·6 | 2·801 | 50·89 | 2·54 | ·128 | 58·3 | 1·143 | 19·60 | 2·66 | ·052 | 60·5 | 2·413 | 39·88 | 7·38 | ·294 |
| " 6th, | 84·0 | 2·543 | 30·27 | 2·55 | ·079 | 57·2 | 2·168 | 37 90 | 2·62 | ·099 | 48·6 | 1·754 | 36·09 | 2·23 | ·080 | 60·7 | 2·106 | 34·69 | 7·43 | ·252 |
| " 7th, | 75·0 | 3·261 | 43·48 | 2·29 | ·098 | 54·7 | 2·478 | 45·32 | 2·51 | ·113 | 44·6 | 1·825 | 40·91 | 2·55 | ·083 | 56 0 | 2·421 | 43·23 | 6·85 | ·296 |
| " 8th, | 86·4 | 4·109 | 47·55 | 2·69 | ·126 | 54·1 | 2·269 | 41·92 | 2·43 | ·104 | 50·5 | 1·937 | 38·35 | 2·36 | ·088 | 60·8 | 2·609 | 42·91 | 7·42 | ·312 |
| " 9th, | 68·7 | 3·751 | 54·71 | 2·61 | ·114 | 57·1 | 2·823 | 49·47 | 2 62 | ·129 | 59 9 | 1·169 | 19·34 | 2·44 | ·053 | 58·7 | 2·435 | 43·01 | 7·22 | ·298 |
| Mean, . . | 74·1 | 3·430 | 46·27 | 2·23 | ·104 | 63·8 | 2·468 | 38·68 | 2·92 | ·113 | 53·3 | 1·543 | 28·95 | 2·44 | 070 | 62·4 | 2·466 | 39 52 | 7·64 | ·301 |

From this table it is seen that hour for hour more urine and phosphoric acid were excreted during the morning period, than either the evening or night, and that the proportion of this latter substance in one thousand cubic centimetres of urine, was greater for the morning urine than for the urine of either of the other periods. Considering that the morning embraced but six hours, whilst the evening and night were each nine hours long, the former period also shows a greater excretion of urine and phosphoric acid, for each pound weight of the body. Indeed, the *absolute* average amount of phosphoric acid in relation to the weight of the body, is much less during the night than the morning, and is but little greater during the evening, notwithstanding the difference in the number of hours.

It would appear from these experiments, that no definite relation exists between the weight of the body and the amount of urine and phosphoric acid excreted, and that the quantity of this latter substance eliminated bears no constant ratio to the amount of urine or to its specific gravity. The investigations are, however, far too meagre to warrant the deduction of definite conclusions on any of these points.

### II.

### EFFECTS OF PHYSICAL EXERCISE.

The physical exercise of this series consisted in the lifting of a weight of one hundred pounds ten feet in a minute, for three periods of fifteen minutes each, at intervals of one hour. This exercise was only employed in the morning, and was in lieu of so much of the time given in the former series to study. In all other respects the course of living was the same as before stated. This series of experiments was continued for five days. The accompanying table exhibits the results.

TABLE III.

| DATE. | MORNING (6 hours). | | | | EVENING (9 hours). | | | | NIGHT (9 hours). | | | | WHOLE DAY. | | | |
|---|---|---|---|---|---|---|---|---|---|---|---|---|---|---|---|---|
| | Quantity of Urine. | Specific Gravity. | Phosph. Acid. | Weight of Body. | Quantity of Urine. | Specific Gravity. | Phosph. Acid. | Weight of Body. | Quantity of Urine. | Specific Gravity. | Phosph. Acid. | Weight of Body. | Quantity of Urine. | Specific Gravity (Mean). | Phosph. Acid. | Weight of Body (Mean). |
| April 10th. | 623·3 | 1023·18 | 32·715 | 195·46 | 525·2 | 1024·25 | 18·534 | 195·40 | 510·5 | 1021·04 | 18·215 | 195·50 | 1659· | 1022·82 | 64·464 | 195·45 |
| " 11th, | 584·9 | 1024·05 | 30·244 | 195·30 | 604·5 | 1024·03 | 22·613 | 195·28 | 539· | 1021·28 | 25·124 | 195·38 | 1728·4 | 1023·12 | 77·981 | 195·32 |
| " 12th, | 645·7 | 1023·20 | 25·187 | 195·15 | 493·5 | 1025·11 | 12·250 | 195·20 | 478·5 | 1022·30 | 17·382 | 195·22 | 1617·7 | 1023·53 | 54·819 | 195·19 |
| " 13th, | 615·5 | 1023·54 | 33 250 | 194·90 | 518· | 1024·51 | 20·327 | 194·87 | 482·7 | 1022·25 | 20·845 | 195·02 | 1616·2 | 1023·43 | 74·422 | 194·93 |
| " 14th, | 580·5 | 1024·80 | 36·185 | 194·82 | 524·6 | 1024·15 | 26·450 | 194·74 | 512·6 | 1021·87 | 24·612 | 194 81 | 1617·7 | 1023·61 | 87·247 | 194·79 |
| Average, | 609·9 | 1023·75 | 31·516 | 195·12 | 533·1 | 1024·41 | 20·034 | 195·09 | 504·6 | 1021·74 | 21·235 | 195·18 | 1647·8 | 1023·30 | 71·786 | 195 13 |

From the examination of this table the influence of the extra amount of physical exercise taken is very readily perceived.

1st. It is seen that in the morning period the quantity of urine, its specific gravity, and amount of phosphoric acid, are very considerably increased over the normal average for this period.

2d. That in the evening there is a reduction of the amount of urine and phosphoric acid, instead of, as in the former series, an increase. The specific gravity of the urine is not so much raised as in the standard series.

3d. That at night the urine and its specific gravity fall to the minimum, whilst the phosphoric acid rises slightly over the evening average.

4th. That for the whole twenty-four hours, as compared with the averages for this period of the preceding series, there is an increase in the quantity of urine, a slight decline in the specific gravity, and a very considerable augmentation of the amount of phosphoric acid excreted.

It is also seen from this table, that the weight of the body is almost invariably highest at night, next highest in the morning, and lowest in the evening.

The comparatively low average specific gravity of the urine for the whole day, was doubtless due to the fact of the employment of the exercise only in the morning. The immediate effect of the physical exertion is seen to have been an increase in the density of the urine. Had the exercise been kept up during the other periods of the day, the result would undoubtedly have been an augmentation for the whole twenty-four hours.

### III.

### EFFECTS OF PHOSPHATE OF SODA.

During this series of investigations I took three hundred grains of crystallized phosphate of soda per day; one hundred at 9 A.M., one hundred at 3 P.M., and one hundred at 10 P.M. At each of these hours, therefore, I ingested nearly twenty grains of phosphoric acid into the system, making sixty grains in the twenty-four hours. This series lasted as the preceding, five days. The conditions of food, exercise, &c., remained as in the first series. The results are embodied in the ensuing table.

TABLE IV.

| Date. | Morning (6 hours). | | | | Evening (9 hours). | | | | Night (9 hours). | | | | Whole Day. | | | |
|---|---|---|---|---|---|---|---|---|---|---|---|---|---|---|---|---|
| | Quantity of Urine. | Specific Gravity. | Phosph. Acid. | Weight of Body. | Quantity of Urine. | Specific Gravity. | Phosph. Acid. | Weight of Body. | Quantity of Urine. | Specific Gravity. | Phosph. Acid. | Weight of Body. | Quantity of Urine. | Specific Gravity (Mean). | Phosph. Acid. | Weight of Body (Mean). |
| April 16th, | 585·6 | 1024·09 | 36·391 | 194·92 | 523·7 | 1025·51 | 40·125 | 194·98 | 429·2 | 1025·40 | 27·896 | 194·89 | 1548·5 | 1025· | 104·412 | 194·93 |
| " 17th, | 627·9 | 1024·11 | 42·587 | 194·83 | 480·5 | 1026·60 | 35·217 | 194·96 | 518·6 | 1025·05 | 29 231 | 194·96 | 1617· | 1025·25 | 107·035 | 194·91 |
| " 18th, | 685·4 | 1024·48 | 47·256 | 194·70 | 527·4 | 1025·38 | 30·625 | 194·81 | 483·6 | 1026·11 | 33·192 | 194·75 | 1696·4 | 1025·32 | 111·073 | 194·75 |
| " 19th, | 592·4 | 1023·80 | 39·110 | 194·51 | 500·2 | 1025 14 | 38·246 | 194·55 | 539·3 | 1023·04 | 25·451 | 194·53 | 1631·9 | 1023·99 | 102·807 | 194·53 |
| " 20th, | 550·5 | 1024·23 | 33·475 | 194·20 | 531· | 1025·30 | 36·351 | 194·32 | 490·1 | 1026·58 | 29·397 | 194·23 | 1577·6 | 1025·37 | 99·223 | 194·25 |
| Average, | 608·3 | 1024·14 | 39·761 | 194·63 | 512·3 | 1025·58 | 36,112 | 194·72 | 492 1 | 1025·23 | 29·033 | 194·67 | 1614·2 | 1024·98 | 104·910 | 194·67 |

From this table it is seen that after the exhibition of phosphate of soda, the following effects ensued:

1st. For the morning period, there was a considerable increase in the quantity of urine, its specific gravity, and amount of phosphoric acid, as compared with the means of the standard series.

2d. For the evening, the quantity of urine was slightly reduced, and the specific gravity and phosphoric acid augmented.

3d. For the night period, the quantity of urine was but slightly affected; the specific gravity and phosphoric acid were, however, increased.

4th. For the whole twenty-four hours, the quantity of urine, its specific gravity, and amount of phosphoric acid, were all increased.

The weight of the body declined steadily during each series of investigations.

Of the sixty grains of phosphoric acid daily ingested, it is probable the whole was not eliminated with the urine. Thus the average normal amount of phosphoric acid daily excreted is seen to have been 59·104 grains, whilst under the administration of the phosphate of soda, it rose only to an average of 104·910 grains, leaving 14·194 grains either discharged with the urine, or retained in the system.

The results obtained by the foregoing investigations differ in several important points from those arrived at by the physiologists before mentioned.

The meagreness of the experiments forbids the deduction of definite conclusions from them, and they are, therefore, only submitted as contributions to a fuller understanding of the subjects to which they pertain.

